# Molecular and pathological subtypes related to prostate cancer disparities and disease outcomes in African American and European American patients

**DOI:** 10.3389/fonc.2022.928357

**Published:** 2022-08-10

**Authors:** Joakin O. Mori, Jason White, Isra Elhussin, Babatunde M. Duduyemi, Balasubramanyam Karanam, Clayton Yates, Honghe Wang

**Affiliations:** ^1^ Department of Biology and Center for Cancer Research, Tuskegee University, Tuskegee, AL, United States; ^2^ Department of Integrative Biosciences, Tuskegee University, Tuskegee, AL, United States; ^3^ College of Medicine and Allied Health Sciences, University of Sierra Leone Teaching Hospital, Freetown, Sierra Leone

**Keywords:** cancer disparities, molecular subtype classification, prognosis, prostate cancer, oncogenic pathways

## Abstract

Prostate cancer (PCa) disproportionately affects African American (AA) men, yet present biomarkers do not address the observed racial disparity. The objective of this study was to identify biomarkers with potential benefits to AA PCa patients. Differentially expressed genes (DEG) analysis coupled with gene set enrichment analysis (GSEA) and leading-edge genes analysis showed that the keratin family of genes, including *KRT8*, *KRT15*, *KRT19*, *KRT34*, and *KRT80*, constituted the single most prominent family of genes enriched in AA compared to European American (EA) PCa cell lines. In PCa patients (TCGA and MSKCC patient cohorts), *KRT8*, *KRT15*, and *KRT19* expression were relatively higher in AA than in EA patients. The differences in the expression of *KRT15* and *KRT19*, but not *KRT8*, were enhanced by Gleason score and ERG fusion status; in low Gleason (Gleason ≤ 6 [TCGA cohort] and Gleason ≤ 7 [MSKCC cohort]), the expression of *KRT15* and *KRT19* was significantly (p ≤ 0.05) higher in AA than in EA patients. Survival analysis revealed that high expression of *KRT15* and *KRT19* was associated with increased risk of biochemical recurrence in low Gleason category patients in the TCGA patient cohort. Interestingly, *KRT15* and *KRT19* expression were also associated with an increased risk of death in the metastatic prostate adenocarcinoma cohort, suggesting the potential to predict the risks of disease recurrence and death in the low Gleason category and advanced disease conditions respectively. Gene set enrichment analysis revealed known oncogenic gene signatures, including *KRAS* and *ERBB2*, to be enriched in patients expressing high *KRT15* and *KRT19*. Furthermore, high *KRT15* and *KRT19* were linked to the basal and LumA PCa subtypes, which are associated with poor postoperative androgen deprivation therapy (ADT) response compared to the LumB subtype. Taken together, the present study identifies genes with high expression in AA than in EA PCa. The identified genes are linked to oncogenic gene signatures, including KRAS and ERBB2, and to basal and LumA PCa subtypes that are associated with poor postoperative ADT response. This study, therefore, reveals biomarkers with the potential to address biomarker bias in PCa risk stratification and/or prognosis.

## Introduction

Prostate cancer (PCa) is the most common cancer and the second leading cause of cancer-related death among men in the United States. African American (AA) men are particularly disproportionately affected; AA men are about twice more likely to be diagnosed with PCa and over two times more like to die from PCa than EA men (1). The underlying cause of PCa health disparity is multifactorial, ranging from molecular differences to the lack of diversity in management strategy. For instance, transmembrane protease, serine 2 (TMPRSS2)-related gene rearrangements are most common in tumors from PCa patients of European ancestry but are significantly less frequent in PCa patients of African and Asian ancestries (2–5). Presently, the management of prostate confined tumors include either active surveillance, radical prostatectomy, or radiation. Active surveillance is recommended for low-risk disease patients: PSA<10 ng/mL, PSA density ≤ 0.15 ng/mL/cm3, clinical-stage ≤ T1c, Gleason sum ≤ 6, positive cores ≤ 2, and cancer involvement per core ≤ 50% (6, 7). However, studies show that active surveillance might not be ideal for some patients, particularly AA men. Studies show AA patients recommended for active surveillance have adverse pathologic features at radical prostatectomy and poorer oncologic outcomes than EA men (6, 8–10). Additionally, the probability of discontinuing active surveillance was higher in black men than in non-black men (11). Furthermore, the disparity in PCa-associated death was observed to be more significant in low-grade (Gleason score ≤ 6) disease patients than in intermediate (Gleason score 7) and high-grade disease (Gleason score ≥8) (10). Different histological, molecular subtypes with racial differences are associated with clinical outcomes have been well accepted in other cancers, like breast, ovarian cancers, etc. This, however, has not been established in PCa. The diverse causes of PCa disparity present a need to diversify management strategies. Thus, proper molecular subtyping would be more relevant to PCa aggressiveness, treatment response, and disparities in PCa. In this report, we aimed to identify biomarkers that may be used in clinical settings for accurate PCa patient risk stratification for a biomarker-guided, personalized treatment approach. Our overall findings demonstrated that cytokeratin 15 (*KRT15*) and *KRT19* are differentially expressed between AA and EA PCa patients; significantly high expression in AA than in EA patients. The findings also linked *KRT15* and *KRT19* expression to the basal and LumA PCa subtypes and further demonstrated that high expression of *KRT15* and *KRT19* was associated with increased risk of biochemical recurrence and reduced overall survival. Our findings may provide new mechanistic insights into PCa disparities and therapeutic approaches.

## Materials and methods

### Cell culture and RNA-seq

African American PCa cell lines RC77T and RC43T (12) along with RC165 were previously established and characterized in our out lab. The cells were cultured in Keratinocyte basal medium supplemented with 10ng/ml EGF and incubated at 37°C, 5% v/v CO_2_. RNA sequencing was isolated from cultured cells using TRIzol^®^ Reagent (Sigma Life Sciences, St. Louis, MO) following the manufacturer’s protocol. Library preparation, quality control, and sequencing of extracted RNA were performed by Novogene Corporation Inc. (Sacramento, CA), with the sequencing data compiled as FastQ files for downstream analysis.

### RNA-sequence analysis, DEG selection and RT-PCR

RNA-Sequence analyses was completed with Partek^®^ Flow^®^ 8.0 (Copyright^®^, 2019 Partek Inc., St. Louis, MO, USA) using default settings. Briefly, RNA FastQ files were obtained from Sequence Read Archive (SRA) using accession numbers [SRR8615579] (MDA PCa 2b, LNCaP, and VCaP), and [SRR10575173] (RWPE-2). The FastQ files for the AA cells line RC77T, RC43T, and RC165T were in-house. After the importation of RNA FastQ files into Partek Flow, raw reads were trimmed with a minimum PHRED quality of 20 and then aligned to hg19 using STAR 2.6.1 (13). Using Partek’s E/M algorithm (14) and RefSeq Transcripts 90 – 2019-5-03, aligned reads were quantified into raw counts. Differential expression analysis of raw counts was completed using DESeq2 3.5 (15). To identify DEGs of interest, a pre-ranked gene list was constructed as previously described in Jaynes et al. (16). After importation into *GSEA_4.1.0.app [build: 27] (* 17, 18), gene set enrichment analysis (GSEA), of biological processes [c5.go.bp.v7.4.symbols.gmt], was performed using the *GSEA Preranked* tool. Finally, the GSEA Leading-edge analysis tool was used to identify the most frequently occurring genes within the 20 gene sets with the highest normalized enrichment score.

The Cancer Genome Atlas (TCGA) program’s PCa (19) and the Memorial Sloan Kettering Cancer Center (MSKCC) PCa cohort (20) data sets, obtained from cbioportal (21, 22), were used to evaluate the differences in gene expression between AA and EA PCa patients. In the TCGA data sets, the gene expression was first compared without patient stratification. After patients were stratified by first, Gleason score (Gleason score ≥ 8 (high-risk), Gleason-score = 7 (intermediate-risk), and Gleason score = 6 (low-risk), and then Gleason score and ERG fusion status before analysis of differences in gene expression. All analyses were performed using RStudio Version 1.4.1103 ^©^ 2009-2021 RStudio. Differences in expression were considered significant if p ≤ 0.05. The MSKCC data set was used to validate gene expression in low Gleason (6 and 7) and ERG fusion negative groups. Both Gleason 6 and 7 were considered low Gleason group in the MSKCC because small sample size.

TRIzol^®^ Reagent (Sigma) was used to isolate RNA, including mRNA from cells. cDNA was obtained from mRNA by reverse transcription using the High-Capacity cDNA Reverse Transcription kit (REF 4374966 or 4368814 by Applied Biosystems) according to the manufacturer’s instruction. Quantitative RT–PCR was performed using PowerUpTM SYBR^®^ Green Master Mix (Applied Biosystems) on a 7500 Fast Real-Time PCR System (Applied Biosystems). Each sample was prepared in triplicate and the housekeeping gene beta-actin was used as an internal control for gene expression normalization.

### Immunohistochemistry

TMA was constructed from the FFPE blocks of representative ACCs using a manual tissue-arraying instrument. TMA tissue sections (5μM) obtained from core biopsies were used to run Immunohistochemistry (IHC). Tissues were incubated for 1 hour at 60°C, followed by deparaffinization in three Xylene baths. Rehydration was done in graded (100%, 95%, and 75%) ethanol concentrations, later transferred to distilled water. Antigen retrieval was performed with 1X IHC Antigen Retrieval Solution 10X High pH (REF 00-4956-58, eBioscience) for 10 minutes at 20 kPa. Endogenous peroxidase was blocked with 3% hydrogen peroxide in 1X PBS IHC Wash Buffer with Tween 20 (PBST) for 5 minutes. Sections were incubated in 3% goat serum for 45 minutes, followed by one-hour incubation with the primary antibody in 1X PBST. After washing twice with 1X PBST, the sections were incubated with peroxidase-labeled secondary antibody for 45 minutes. The staining was visualized with 3, 3’-diaminobenzidine (DAB) as chromogen. Slides were counterstained with hematoxylin, dehydrated, and then mounted. All slides were interpreted by an experienced pathologist. For all IHC stains, tumors were scored as 0 (negative), 1+ (weakly positive), 2+ (moderate staining), 3+ (strong staining). The H-score was determined by adding the results of multiplication of the percentage of cells with staining intensity ordinal value with highest 300 possible values. *H-Score*=1∗(% *cells* 1+)+2∗(% *cells* 2+)+3∗(% *cells* 3+). The work was carried out in accordance with the guidelines approved by Tuskegee University Institutional Review Board (IRB).

### Pathway and function enrichment analysis

The oncogenic and immunogenic gene signature associated with the expression of the DEGs of interest was evaluated in the TCGA PCa cohort. mRNA expression data for the cohort was obtained from cBioportal (21, 22). To identify oncogenic and immunogenic gene signatures associated with gene expression, DEG analysis was performed using iDEP.92 (23). The results of the differentially expressed genes presented as LOG_2_FC (fold change) were exported as.csv files for downstream analysis, including gene set enrichment and leading-edge gene analyses to identify enriched oncogenic/immunogenic and leading-edge genes, respectively. For gene set enrichment analysis, a pre-ranked gene list was constructed as previously described (16). After importation, of the pre-ranked gene list into the *GSEA_4.1.0.app [build: 27] (* 17, 18), oncogenic and immunogenic gene set enrichment analysis were performed using the *GSEAPreranked* tool (default setting) with either the c6.all.v7.4.symbols.gmt [Oncogenic signature] and the c7.all.v7.4.symbols.gmt [Immunogenic signature] gene sets databases, respectively. Finally, the GSEA Leading-edge analysis tool was used to identify the most frequently occurring genes within the 20 gene sets with the highest normalized enrichment score.

### Correlation of DEGs with PAM50 subtypes

To evaluate the association of gene expression with PCa subtypes, including LumA, LumB, and Basal subtypes, we used the PCa Transcriptome Atlas (PCTA) web tool (24) was used to. The analyses were based on the PCTA dataset using the One-way ANOVA test. Differences in expression between the groups were considered significant if p ≤ 0.05.

### Survival outcome analysis

The *Kaplan-Meier Plotter (* 25) was used to evaluate the associations of the expression of DEGs of interest with disease outcomes, including biochemical recurrence (BCR) and overall survival (OS). Patients were split by either the Trichotomization or the Auto select best cutoff tool. The associations of the expression of the DEGs of interest with biochemical recurrence and overall survival were evaluated in the TCGA PCa and SU2C/PCF Dream Team cohorts, respectively. Association with biochemical recurrence was assessed by the Gleason category, including 6, 7, and ≥ 8. The association with overall survival was assessed by follow-up period, including 24, 30, and 60-month follow-up periods. In addition to the association with individual gene expression, the impact of identified DEGs as a panel on overall survival was also evaluated. Association with disease outcome was considered significant if HR (hazard ratio) or p-value was ≥2 or ≤0.05.

### Association of DEGs with immune cells infiltration

To quantify the tumor-associated immune cell populations, we used the Tumor Immune Estimation Resource–TIMER2.0 (26) to analyze the association of gene expression with the infiltration of the immune cells: CD8+ T cells, B cells, and macrophages. Associations were considered significant if ≥50% of the algorithms used in TIMER2.0 predicted a statistically significant association.

## Results

### Genes differentially expressed between African American and European American prostate cancer cells

**Figure 1:**
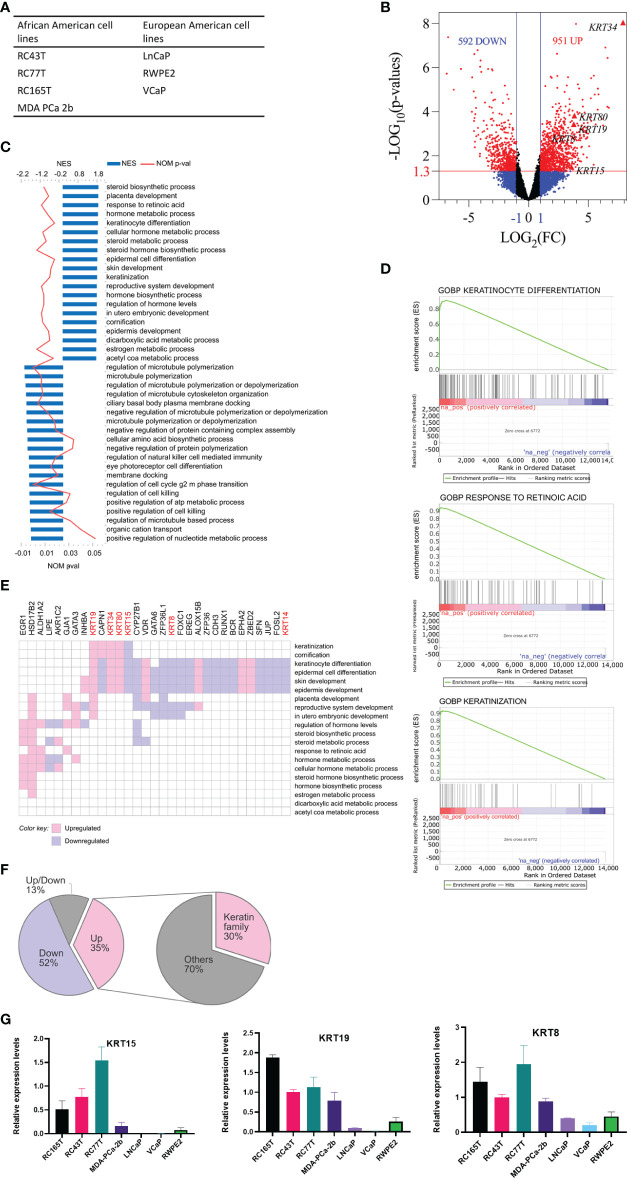
Analysis of differentially expressed genes (DEG) between AA and EA PCa cell lines. **(A)** AA and EA PCa cell lines. **(B)** volcano plots of DEGs; genes were differentially expressed if FC ≥ 2 (LOG_2_(FC) ≥ 1) and p ≤ 0.05 (-LOG_10_P = 1.3). **(C)** GOBP (gene ontology biological processes) gene sets enriched in AA PCa cell lines relative to EA PCa cell lines. **(D)** representative gene set enrichment plots. **(E)** leading-edge genes in top 20 positively enriched gene sets. **(F)** proportions of leading-edge genes; the keratin family of genes constituted the single most prominent family of DEGs enriched in AA. **(G)** Validation of RNA-seq data by RT-qPCR. The expression of selected DEGs in cancer cell lines quantified by qRT-PCR were shown.

We performed RNA sequencing analysis, comparing AA PCa cell lines, RC77T, RC165T, RC43T, and MDA PCa 2b with the EA PCa cell lines LNCaP, RWPE2, and VCaP (**Figure 1A**). Differential gene expression (DEG) analysis revealed 592 significantly downregulated genes (p ≤ 0.05) and 951 significantly upregulated genes (p ≤ 0.05) in the AA cell lines compared to the EA cell lines (**Figure 1B**). Gene set enrichment analysis showed that the AA cell lines were positively enriched in 273 gene sets and negatively in 23 gene sets (**Figure 1C**), including gene sets associated with keratinocyte differentiation, response to retinoic acid, and keratinization (**Figure 1D**). Furthermore, Leading-edge analysis, revealed genes, including *KRT8*, *KRT15*, *KRT19*, *KRT34*, and *KRT80*, in the cytokeratin family of genes to be the most common among the leading-edge genes in the top 20 most common genes (**Figures 1E, F**). The differences in expression of the leading edge cytokeratin family genes observed in the RNA seq data was confirmed by Real-time PCR analysis (**Figure 1G**). *KRT15, KRT19 and KRT8* were amplified and the expression were significiantly higher in the AA cancer cell lines compared to EA cell lines. *KRT34* and *KRT80* expression levels were too low or were hardly detected in all the prostate cancer cell lines (Data not shown). Additional genes identified, including *HSD17B2*, *CYP27B1*, *ZFP36L1*, *EGR1*, *VDR*, *CAPN1*, *FOXC1*, *EREG*, *GATA6*, *ALOX15B*, *LIPE*, *GJA1*, *ZFP36*, *CDH3*, and *RUNX* (**Figure 1E**) have been implicated in PCa progression (27–38).

### Expression profile of differentially expressed keratins in patient populations

**Figure 2:**
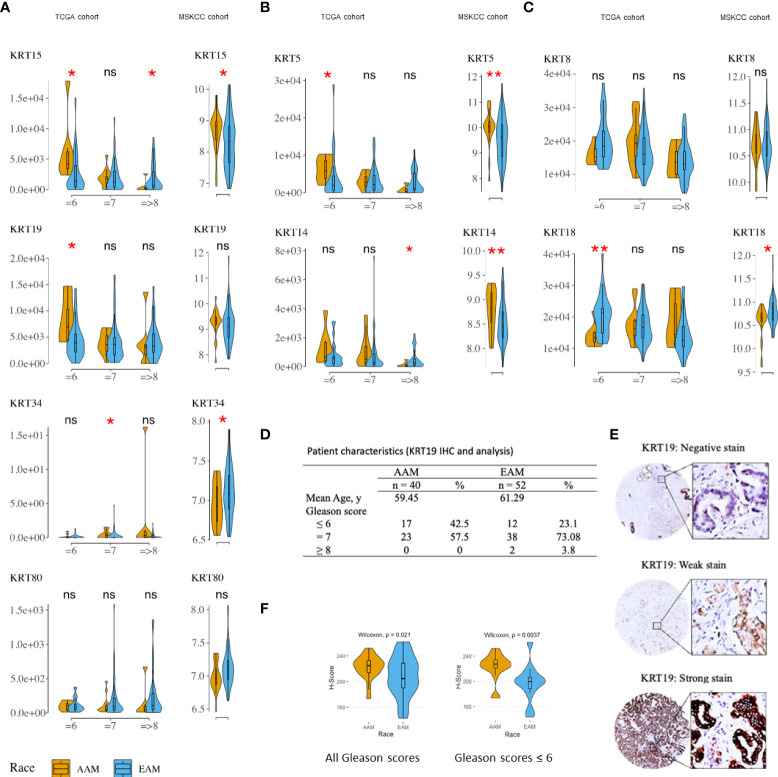
Distribution and median expression levels of keratins in AA and EA PCa patients (TCGA [ERG fusion negative] and MSKCC cohorts). **(A)** differentially expressed keratins (excluding *KRT8* – see C) in the TCGA (left column) and MSKCC (right column) cohorts. **(B)** basal cell keratins (*KRT5* and *KRT14*). **(C)** luminal cell keratins (*KRT8* and *KRT18*). Statistically significant differences in gene expression were determined using the nonparametric Wilcoxon-Mann-Whitney test: *p ≤ 0.05; **P ≤ 0.01. TCGA patients were stratified by Gleason (risk) categories; that is Gleason = 6 (low risk), Gleason = 7 (intermediate risk), and Gleason => 8 (high risk) categories. **(D)** Clinicopathological characteristics of prostate cancer patients in the deidentified prostate tumor cohort. **(E)** Immunohistochemical staining of KRT19 in prostate cancer tissues. Representative images of KRT19 negative, weak or strong staining. **(F)** boxplot of KRT19 H-Scores illustrating significant differences in AA Vs. EA prostate cancer patients. * P values < 0.05 were considered statistically significant. All the patients (upper panel) and patients with Gleason ≤6 (lower panel). ns, not significant.

Since the cytokeratin family was the most enriched gene set in the AA cancer cells, we sought to determine if the same trend could be observed in PCa patients. For this analysis, we used the TCGA and MSKCC PCa patient cohorts. The characteristics of the cohorts were described previously in other studies (19, 20). The expression of *KRT8*, *KRT15*, and *KRT19* was relatively higher in AA compared to EA PCa patients in both the TCGA (**Supplementary Figures 1A, C**) and MSKCC cohorts (**Figure 2A**); the difference in expression of *KRT15* was statistically significant (p ≤ 0.05) in the MSKCC (**Figure 2A**). The difference in expression of identified *KRTs* between AA and EA patients was influenced by the Gleason score and ERG fusion status. For instance, *KRT19* expression was significantly (p ≤ 0.05) higher in AA in Gleason six patients but was not significant in EA in Gleason seven and Gleason ≥8 patients; a similar trend was observed for *KRT15* (**Supplementary Figure 1B**). Additionally, the expression of both *KRT15* and *KRT19* was significantly higher in AA than in EA Gleason six, ERG fusion negative patients in the TCGA cohort (**Figure 2A**). The expression of *KRT80* was lower in AA patients in both the TCGA (p ≤ 0.05) and MSKCC cohorts and the differences in expression seem not to be influenced by Gleason or ERG fusion status (**Supplementary Figure 1B** and **Figure 2A**). The expression of *KRT34 and KRT80* were too low for us to meaningfully evaluate differences in expression between AA and EA patients by the Gleason score and ERG fusion status (**Figure 2A**). The cytokeratin genes *KRT5*, *KRT14*, *KRT8*, and *KRT18* have been used by multiple groups to distinguish prostatic basal and luminal epithelial cells (39, 40). KRT5 and KRT14 are enriched in basal epithelial cell types, while KRT8 and KRT18 are enriched in the luminal epithelial cell types. In the present study, we also sought to determine if there were differences, between AA and EA PCa patients, in the expression of the epithelial basal and luminal cell cytokeratins. Our analysis showed that the epression of basal cell *KRT5* and *KRT14* were significiantly higher in AA than in EA in the MSCKCC cohort bu only slightly higher in TCGA (Gleason six and ERG fusion negative) cohort, similar to that of *KRT15* and *KRT19* expression (**Figure 2B**). Luminal markers KRT8 and KRT18 expression had no difference in AA and EA patients in the MSCKCC and TCGA cohorts (**Supplementary Figure 1C**) and only KRT18 was slightly higher in EA than in AA MSCKCC cohort and in TCGA Gleason six and ERG fusion negativepatients (**Figure 2C**). To further validate the expression levels of newly identified pivotal and consistant DEGs in AA and EA PCa, KRT15 and KRT19 protein expression levels were validated by Immunohistochemistry (IHC) in prostate tumor samples (**Figure 2D**). The staining intensities of KRT19 were defined as negative, weak and strong staining (**Figure 2E**). KRT19 expression H-scores (**Figure 2F**) were significiantly higher in AA cancer patients compared with EA prostate cancer patients (upper panel, Wilcoxon test: p<0.05). The expression H-scores were significiantly higher in AA and lower in EA Gleason score 6 patients, too (**Figure 2F**, lower panel, Wilcoxon test: p<0.01). However, KRT15 expression levels were not statistically significant in AA cancer patients compared with EA prostate cancer patients (data not shown).

### *KRT15* and *KRT19* expressions correlated with Basal and LumA prostate cancer subtypes

**Figure 3:**
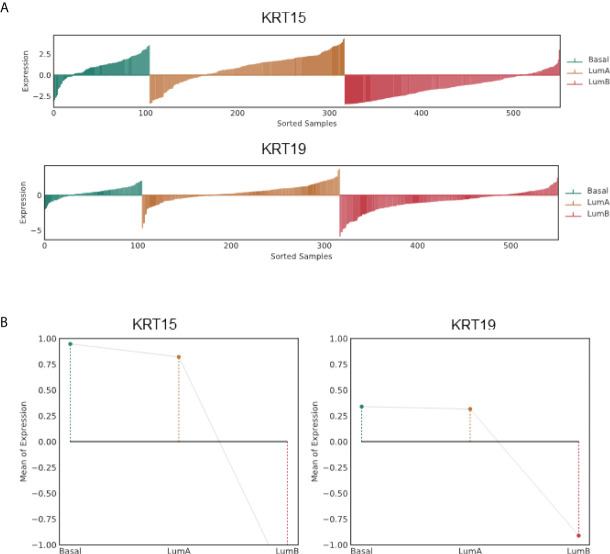
Association of *KRT15* and *KRT19* expression with PCa subtypes in the TCGA PCa patient cohort. **(A)** Lollipop plots. **(B)** Lineplots of mean trends. All analyses were performed in PCTA (24) using the default setting.

The PAM50 PCa subtypes including Basal, LumA, and LumB subtypes have been implicated in postoperative ADT response; Basal and LumA respond poorly to postoperative ADT compared to the LumB subtype. Using the PCa Transcriptome Atlas (PCTA) (24), we evaluated the association of *KRT15* and *KRT19* expression with the PAM50 PCa subtype. The expression of both *KRT15* and *KRT19* positively correlated with Basal and LumA PCa subtypes, and negatively with LumB PCa subtype (**Figure 3** and **Supplementary Figure 2**), suggesting a positive correlation between *KRT15* and/or *KRT19* expression and poor response to postoperative ADT treatment.

### Association of identified cytokeratin with disease outcomes

**Figure 4:**
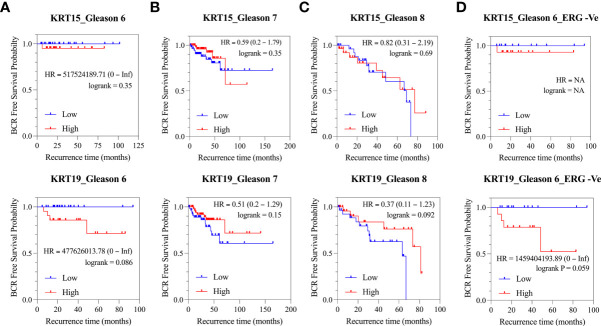
Association of *KRT15* and *KRT19* expression with risk of BCR in PCa patients (TCGA cohort). **(A)** Gleason six patients’ category. **(B)** Gleason seven patients’ category. **(C)** Gleason 8+ patients’ category. **(D)** Gleason six and ERG fusion negative patients’ category.

Since the expression pattern *KRT15* and *KRT19* was consistent across both the TCGA and MSKCC patient cohorts, we sought to evaluate how *KRT15* and *KRT19* expression levels correlate with disease prognosis in the TCGA patient cohort. Patients were trichotomized into low, intermediate, and high *KRT15* or *KRT19* expression, and the risk of BCR in the high expression group was compared to the low expression group. High expression of *KRT15* (HR = 517524189.71 [0 – Inf]; p = 0.35) or *KRT19* (HR = 477626013.78 [0 – Inf]; p = 0.086) was associated with a reduced probability of BCR free survival in the Gleason 6 patients (**Figure 4A**). Furthermore, the separation between the BCR risk curve of the high *KRT19* expression group and the low *KRT19* expression group was greater (HR = 1459404193.89 [0 – Inf]; p = 0.059) in Gleason 6 and ERG fusion negative PCa patients (**Figure 4D**), suggesting the association between *KRT19* expression and risk of BCR is influenced by ERG fusion status. There was no significant association between *KRT15* or *KRT19* expression and risk of BCR **Figures 4B, C**).

**Figure 5:**
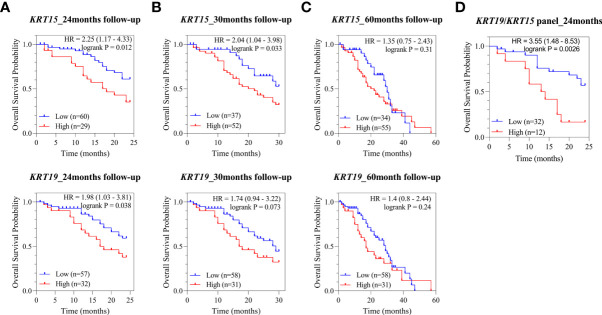
Association of *KRT15* and *KRT19* expression with overall survival. **(A)** 24-months follow-up. **(B)** 30-months follow-up. **(C)** 60-months follow-up. **(D)** *KRT15*/*KRT19* panel.

In PCa, metastasis coupled with the development of castration resistance is the leading cause of death. Therefore, we next assessed the correlation of *KRT15* and *KRT19* expression with overall survival in the Metastatic Prostate Adenocarcinoma (SU2C/PCF Dream Team) patient cohort. High expression of both *KRT15* and *KRT19* was associated with a reduced probability of overall survival at both 24 and 30 months (**Figures 5A, B**). *KRT15* was statistically significant at both 24 (HR = 2.25 [1.17 – 4.33]; p = 0.012) and 30 months (HR = 2.04 [1.04 – 3.98]; p = 0.033), while the association with *KRT19* was statistically significant (HR = 1.98 [1.03 – 3.81]; p = 0.038) at 24-months follow-up and diminished after 30-months (**Figure 5C**). However when we combined, *KRT15* and *KRT19*, both were better at predicting overall survival (HR = 3.55 [1.48 – 8.53]; p = 0.003; **Figure 5D**) than either *KRT15* or *KRT19* alone. Taken together, the present result suggests both *KRT15* and *KRT19* could be novel prognosis markers in predicting overall survival in AA PCa patients.

### Oncogenic and immunogenic gene signatures associated with *KRT15* and *KRT19* expression

**Figure 6:**
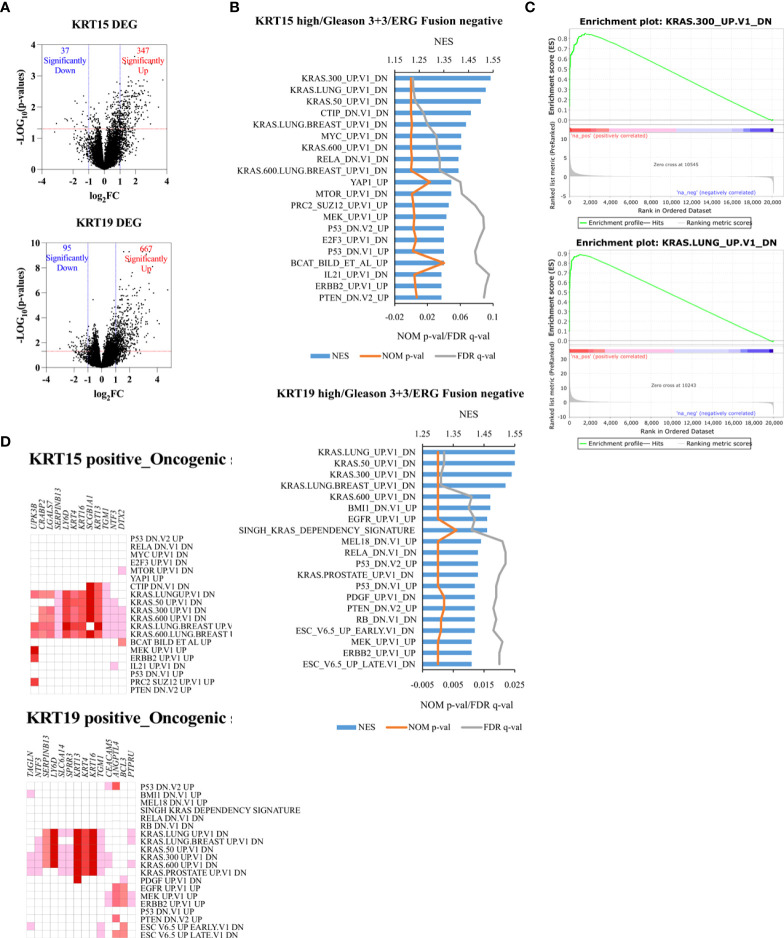
Unique oncogenic gene signature associated with *KRT15* and *KRT19* expression in ERG fusion negative and Gleason 3 + 3 patients. Patients were trichotomized by gene expression into T1 (low expression), and T3 (high expression), and differences in gene expression were determined in low versus high expression patients. **(A)** DEG in high versus low *KRT15* and *KRT19* expression. **(B)** oncogenic gene sets positively enriched in high versus low *KRT15* and *KRT19* expression. **(C)** representative enrichment plots. **(D)** Leading-edge genes.

To identify the functional or targetable gene signatures associated with *KRT15* and *KRT19* expression, ERG fusion negative patients (TCGA cohort) who presented with low Gleason were classified by tertile. Patients in the lower tertile were considered KRT19 negative, while those in the higher tertile were considered KRT19 positive. Differential gene expression analysis revealed 347 genes to be upregulated in KRT15 positive patients, while 37 genes were downregulated; in KRT19 positive versus KRT19 negative patients, 667 genes were upregulated, while 95 genes were downregulated (**Figure 6A**).

**Figure 7:**
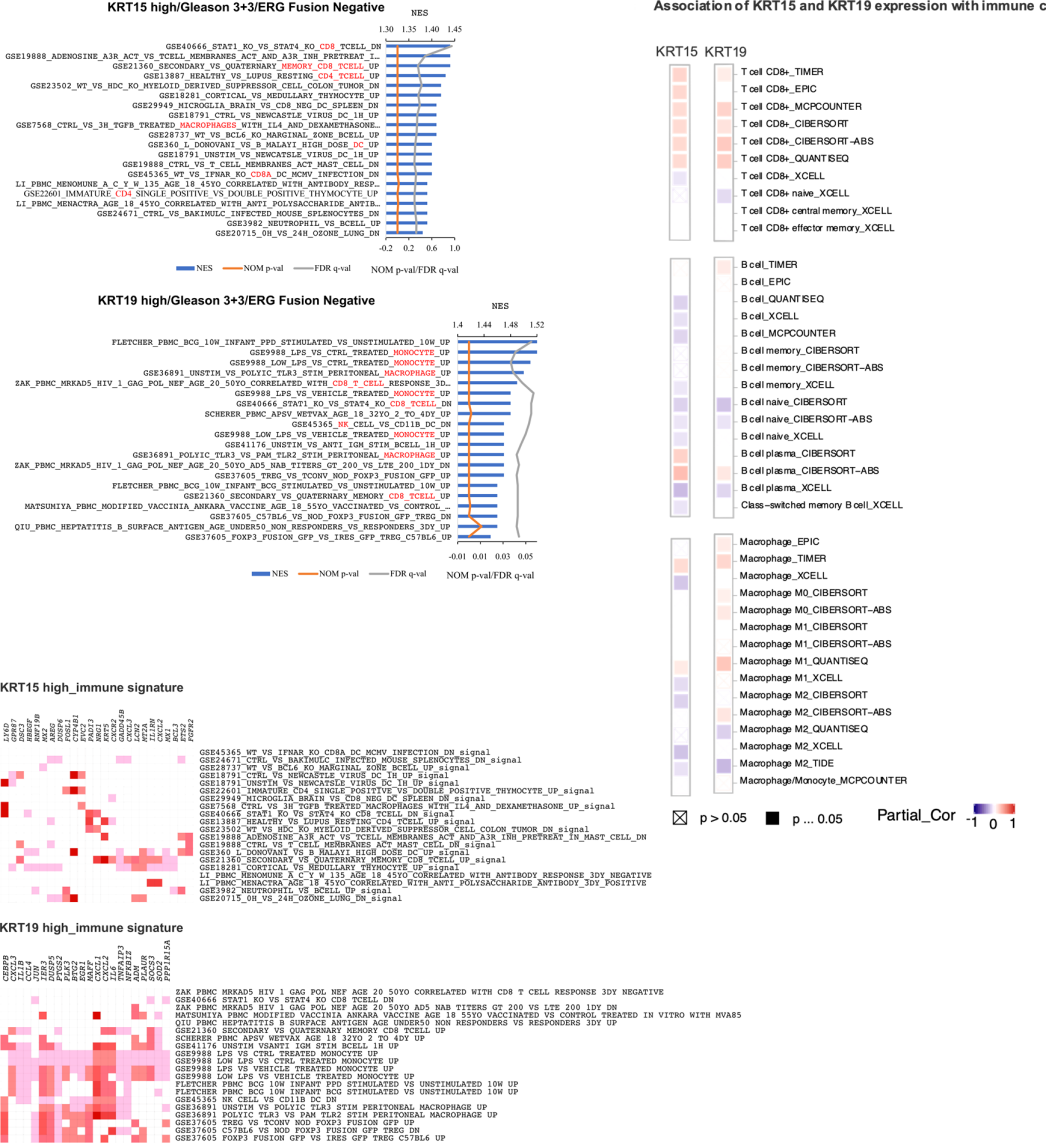
Immunogenic gene signatures associated with *KRT15* and *KRT19* expression in ERG fusion negative and Gleason 3 + 3 patients. **(A)** gene sets positively enriched in patients with high expression of *KR15* (top) and *KRT19* (bottom). **(B)** leading-edge genes associated with gene sets positively enriched in patients with high expression of *KRT15* (top) and *KRT19* (bottom). **(C)** immune cells infiltration associated with *KRT15* and *KRT19* expression. Squares with a cross indicate non-significant associations (p > 0.05), solid square indicates significant associations (p ≤ 0.05), and purple-red is association gradient (purple is for negative association and red for positive association).

Gene set enrichment analysis revealed gene signatures associated with common cancer-related genes including *KRAS*, *PTEN*, *ERBB2*, and *P53* to be significantly (NOM p-val < 0.05 at FDR < 25%) enriched in both KRT15 positive and KRT19 positive patients (**Figures 6B, C**). Leading-edge analysis revealed *UPK3B*, *CRABP2*, *LGALS7*, *SERPINB13*, *LY6D*, *KRT4*, *KRT16*, *SCGB1A1*, *KRT13*, *TGM1*, *NTF3*, and *DTX2* as the leading genes (present in at least 6 gene sets) in the KRT15 positive patients; *TAGLN*, *NTF3*, *SERPINB13*, *LY6D*, *SLC6A14*, *SPRR3*, *KRT13*, *KRT4*, *KRT16*, *TGM1*, *CEACAM5*, *ANGPTL4*, *BCL3*, and *PTPRU* were the top leading genes (present in at least 5 gene sets) in KRT19 positive patients (**Figure 6D**). Eight of the leading-edge genes including *SERPINB13*, *LY6D*, *KRT4*, *KRT16*, *SCGB1A1*, *KRT13*, *TGM1*, and *NTF3* were common to KRT15 positive and KRT19 positive patients. Furthermore, gene sets associated with the activation, inactivation, or functions of CD8+ T cells, B cells, Dendritic cells, CD4+ T cells, and macrophages were enriched in KRT15 positive patients (**Figure 7A**). In KRT19 positive, enriched gene sets included those associated with the activation, inactivation, or functions of natural killer cells, Treg cells, and monocytes in addition to CD8+ Tc cells, B cells, and macrophages (**Figure 7A**). Leading-edge analysis revealed genes including *LY6D*, *GPR87*, *DSC3*, *HBEGF*, *MX2*, *AREG*, *DUSP6*, *FOSL1*, *CYP4B1*, *EVC2*, *PADI3*, *NRG1*, *KRT5*, *CXCR2*, *GADD45B*, *CXCL3*, *LCN2*, *MT2A*, *IL1RN*, *CXCL2*, *MX1*, *BCL3*, *ETS2*, and *FGFR2*; only *CXCL2* and *CXCL3* were common to both KRT15 and KRT19 positivity (**Figure 7B**).

The activation of CXCL2 and its associated receptor CXCR2 by KRAS signaling is thought to suppress immune response and promote tumor proliferation (41, 42). Immune suppression is critical for tumor cell survival and progression (43). In the present study, using TIMER2.0 (26), we estimated the association of *KRT15* and *KRT19* expression with the infiltration of the immune cells (**Figure 7C**). The associations were considered significant if ≥50% of the algorithms used in TIMER2.0 predicted a statistically significant association. There was a significant positive association of both *KRT15* and *KRT19* expression with CD8+ T cell infiltration. On the other hand, there was a significantly negative association between B cell infiltration and *KRT15*, but not *KRT19* expression. Macrophage, particularly M0 macrophage infiltration, was positively associated with *KRT19* expression, but not *KRT15* expression.

## Discussion

In the United States, PCa disproportionately affects AA men; compared to EA men, AA men are more likely to be diagnosed and to die from PCa (1). While the cause of PCa disparity is multifactorial, mischaracterization of risks of PCa progression, leading to erroneous treatment recommendations, may be a contributing factor. For instance, active surveillance is the treatment option for many men with low-risk PCa. However, more African American men with early-stage cancer may harbor more aggressive disease and are more likely to die from PCa than other patients and may not be good candidates for active surveillance (6, 8–11). In the present study, we identified gene sets associated with the development and differentiation of epithelial and epidermal cells; and hormone production, function, and metabolism positively enriched in AA PCa cell lines relative to EA PCa cell lines. Leading-edge gene analysis revealed gene sets including cytokeratin (*KRT*) genes: *KRT19*, *KRT8*, *KRT34*, *KRT80*, *KRT15*, and *KRT14* as the most prevalent family of genes in the top 20 most common genes. Other leading-edge genes included *HSD17B2*, *CYP27B1*, *CYP27B1*, *ZFP36L1*, *EGR1*, *VDR*, *CAPN1*, *FOXC1*, *EREG*, *GATA6*, *ALOX15B*, *LIPE*, *GJA1*, *ZFP36*, *CDH3*, and *RUNX*.

Cytokeratins are intermediate filaments involved in normal cell function and also associated with diseased conditions (44). Normal adult prostatic epithelium consists of basal, secretory luminal, and rare neuroendocrine and intermediate cells, which could be classified by cytokeratin and other differentiation markers. Luminal cells are the majority of the prostate epithelia and carry out the secretory function. The low-molecular-weight cytokeratins KRT8 and KRT18 are typical lineage markers for the luminal cells. The basal epithelial subpopulation expressed the classical high-molecular-weight basal cell markers KRT5, KRT14 and p63. In addition to a very small fraction of the epithelial cells called neuroendocrine (NE) cells (express a variety of NE markers (including chromogranins, synaptophysin, and CD56), there is also a population of transit-amplifying or intermediate prostate epithelial cells. These cells co-express markers of both the basal and luminal epithelial cell markers (KRT5, KRT14, KRT8, KRT18, KRT19, p63 and GSTpi). These rare rapid amplifying prostatic intermediate epithelial cells are proposed to be derived from urogenital or basal progenitor/stem cell population and could differentiate into luminal cells expressing KRT8/18. Lineage plasticity is the ability of cells to trasform from one developmental lineage to another, which is essential for embryonic development, tissue repair and maintenance of homeostasis. This highly regulated cell differentiation process is also considered as a source of intratumoural heterogeneity when cancer cells adapt to tumour microenvironment, lineage plasticity can promote tumor progression, metastasis and therapy resistance (39, 40). Instead of undergoing normal differentiation to form the functional prostate, a subset of cells may arrest at an early stage and show aberrant differentiation causing disruption of the precise signaling pathways, which are critical for prostate morphogenesis during early development ultimately resulting in carcinogenesis. The underling mechanisms for increased intermediate epithelial cell population among AA PC patients remain unclear. Answers may lie in varying social conditions, underlying genetic factors, or unidentified biological factors. On the other hand, cytokeratins are implicated in tumorigenesis, drug responsiveness, cancer cell invasion, and metastasis; and are helpful cancer diagnostic and prognostic markers (44–54). In PCa, expression of *KRT8*, *KRT18*, and *KRT19* by tumor cells disseminated to the bone, is associated with a worse prognosis (49); *KRT18* and *KRT5* expression correlates with metastases and hormone-escaped prostate carcinomas, respectively (55). Our findings demonstrate that *KRT15* and *KRT19* are differentially expressed in a Gleason score and ERG fusion status manner, with fusion negative AA patients expressing higher levels of both *KRT15* and *KRT19*. Elevated *KRT15* and *KRT19* expression were also associated with an increased risk of biochemical recurrence in low Gleason score patients, more so in ERG fusion negative patients. Similarly, high expression of *KRT19*, or *KRT15* was associated with worse survival in a metastatic prostate adenocarcinoma patient cohort (SU2C/PCF Dream Team) regardless of ERG fusion status. Similar findings were reported in breast cancer and hepatocellular carcinoma; KRT19 expression correlated with poor prognosis in breast cancer (56) and predicted early postoperative recurrence in hepatocellular carcinoma (57). Interestingly, in clear cell renal cell carcinomas, the detection of KRT19 along with KRT7 was associated with better clinical outome (44). Elucidating how other signaling pathways and developmental regulators are integrated to modulate prostate organogenesis and differentiation will be of particular relevance for understanding their roles in prostate cancer. For example, mechanisms that drive progenitor cell plasticity in the context of epithelial differentiation and repair could also play a role in prostate tumor plasticity in mediating resistance to targeted cancer therapies.

In this study, our investigation identified a two-gene signature that accurately stratified cancer aggressiveness and provide biological measures indicating the likelihood of a more aggressive disease for AA patients newly diagnosed with localized cancers. Over the years, several prognostic tools for PCa have been developed including serum (4K, phi), urine (Progensa, T2-ERG, ExoDx, SelectMDx), and tissue-based bioimarkers (ConfirmMDx, Prolaris, Oncoytype DX, Decipher) (58). However, these markers do not account for differences associated with racial disparities in PCa. In our study, high expression of both *KRT15* and *KRT19* in low-risk ERG fusion negative patients was associated with the enrichment of common cancer-associated gene signatures, especially KRAS. The aberrant activation of KRAS signaling is a common driver of tumor development and progression in different types of cancers, including pancreatic cancer (59), non-small-cell lung cancer (60–63), colorectal cancer and melanoma (64), and pancreatic cancer (65). KRAS signaling is also thought to activate CXCL2 and its associated receptor CXCR2 resulting in suppressed immune response and tumor proliferation (41, 42). Immune suppression is critical for tumor cell survival and progression (43). The present study also shows high expression of *KRT15* and *KRT19* positively correlated with the PAM50 Basal and LumA phenotype, but negative with the LumB phenotype. The Basal and LumA have been shown to respond poorly to postoperative ADT compared to the LumB subtype (66, 67). Taken together, our study illustrates the potential of *KRT15* and *KRT19* as a PCa prognostic markers for patients who present with low Gleason, particularly African American patients. Although a limitation of our study design is the low number of patients, particularly AA and lack of clinical data, such as biochemical recurrence and overall survival for some patients, further validation with larger sized patient cohorts and mechanistic studies are needed to verify these findings. It’s still worthnoting that current findings highlight the value of developing prognostic tools that can distinguish aggressive tumors vs indolent tumors particularly in low risk AA prostate cancer patients.

## Data availability statement

The original contributions presented in the study are publicly available. This data can be found here: https://www.ncbi.nlm.nih.gov/geo/; GSE203338.

## Author contributions

HW and CY conceived the idea for the study and supervised the work. JM, HW, and BK involved in planning the project, carried out the experiments and interpreted the results. JM, JW, IE, and BD performed data and statistical analysis. JM, HW, CY and JW were involved in writing the manuscript with input from all authors. All authors contributed to the article and approved the submitted version.

## Funding

This work was supported by US National Institutes of Health (NIH) grants U54-MD007585-26 (NIH/NIMHD, CY; HW), U54 CA118623 (NIH/NCI, CY), and Department of Defense Grant (PC170315P1, W81XWH-18-1-0589, CY).

## Conflict of interest

CY is a shareholder in Riptide biosciences and is a consultant in QED Therapeutics, Riptide Biosciences, and Amgen.

The remaining authors declare that the research was conducted in the absence of any commercial or financial relationships that could be construed as a potential conflict of interest.

## Publisher’s note

All claims expressed in this article are solely those of the authors and do not necessarily represent those of their affiliated organizations, or those of the publisher, the editors and the reviewers. Any product that may be evaluated in this article, or claim that may be made by its manufacturer, is not guaranteed or endorsed by the publisher.

## Supplementary material

Supplementary Figure 1 Expression profile of keratins in all race AAM and EAM patients. **(A, B)** differentially expressed keratins (**A** no patient stratification applied; **B** patients stratified by Gleason score category). **(C)** epithelial basal and luminal cell keratins in all race annotated patients.

Supplementary Figure 2 Association of *KRT15* and *KRT19* expression with Basal, LumA, and LumB PCa subtypes (PCTA dataset) (24). **(A)** Lollipop plots. **(B)** Lineplots of mean trends. All analyses were performed in PCTA (24) using the default setting.
