# Supplementary figures and images for: Molecular and pathological subtypes related to prostate cancer disparities and disease outcomes in African American and European American patients
Source: Front Oncol. 2022 Aug 10;12:928357. doi: 10.3389/fonc.2022.928357 (PMC9399459; doi:10.3389/fonc.2022.928357)

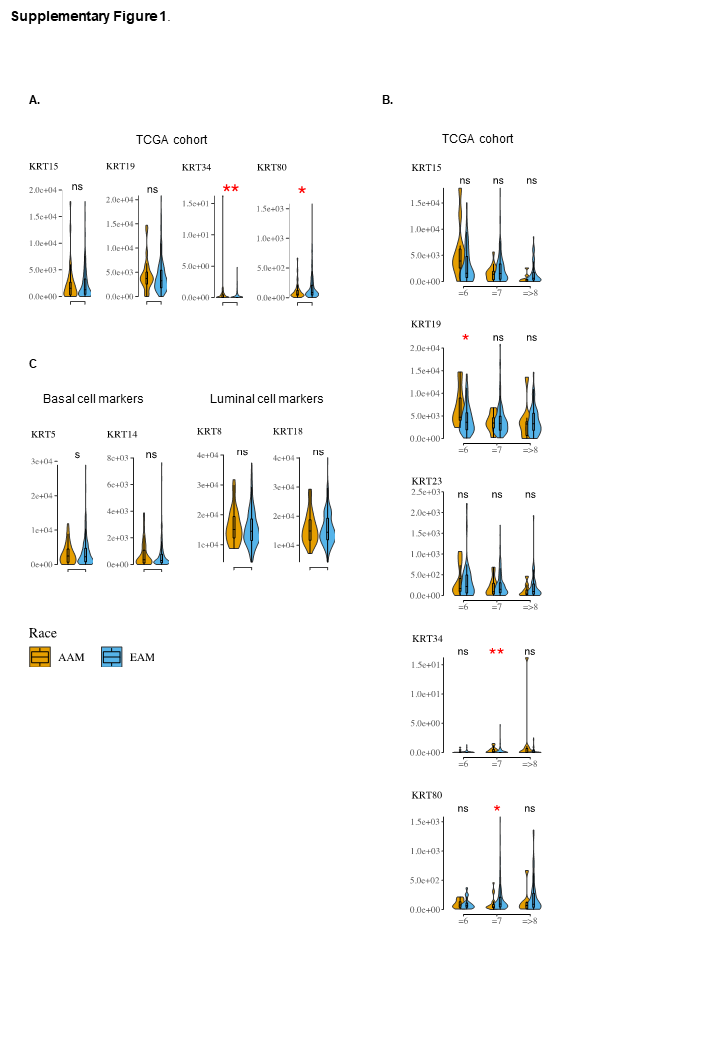

Supplement: Supplementary Figure 1 — Expression profile of keratins in all race AAM and EAM patients. (A, B) differentially expressed keratins (A no patient stratification applied; B patients stratified by Gleason score category). (C) epithelial basal and luminal cell keratins in all race annotated patients. [file Image_1.tif]

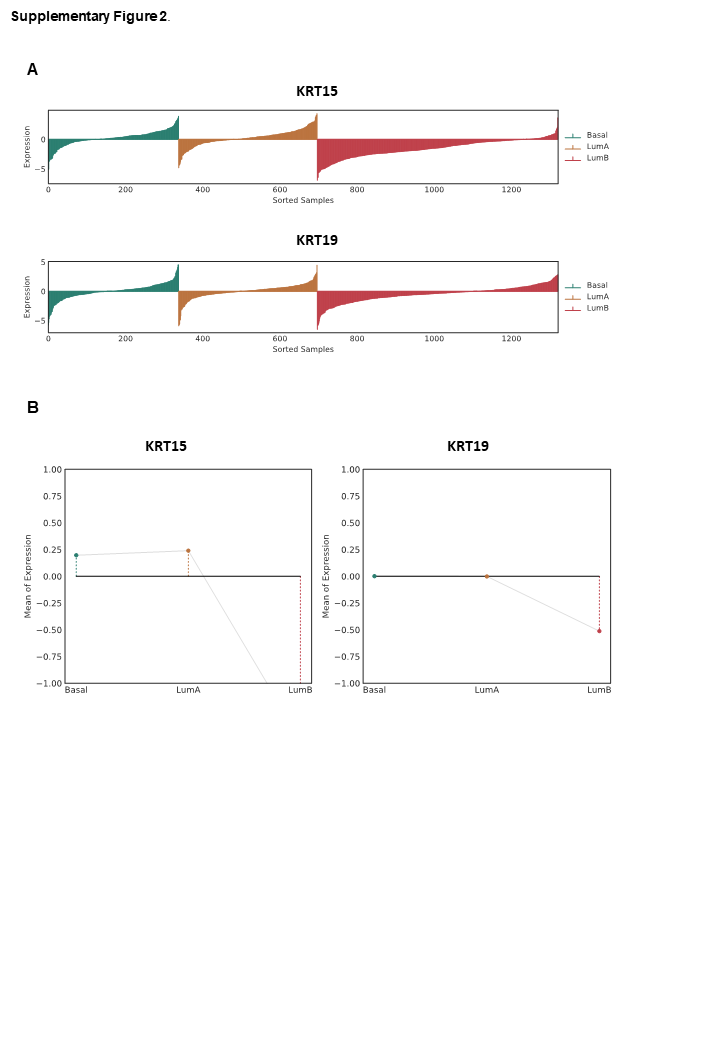

Supplement: Supplementary Figure 2 — Association of KRT15 and KRT19 expression with Basal, LumA, and LumB PCa subtypes (PCTA dataset) (24). (A) Lollipop plots. (B) Lineplots of mean trends. All analyses were performed in PCTA (24) using the default setting. [file Image_2.tif]
